# Effects of Propofol Treatment in Neural Progenitors Derived from Human-Induced Pluripotent Stem Cells

**DOI:** 10.1155/2017/9182748

**Published:** 2017-10-08

**Authors:** Bo Long, Shenglan Li, Haipeng Xue, Li Sun, Dong H. Kim, Ying Liu

**Affiliations:** ^1^Vivian L. Smith Department of Neurosurgery, McGovern Medical School, The University of Texas Health Science Center at Houston, Houston, TX, USA; ^2^Department of Anesthesiology, Shengjing Hospital, China Medical University, Shenyang, China; ^3^Center for Stem Cell and Regenerative Medicine, The University of Texas Health Science Center at Houston, Houston, TX, USA; ^4^Department of Oncology, Renji Hospital, Shanghai Jiao Tong University School of Medicine, Shanghai, China; ^5^Senator Lloyd and B.A. Bentsen Center for Stroke Research, The Brown Foundation Institute of Molecular Medicine, The University of Texas Health Science Center at Houston, Houston, TX, USA

## Abstract

Propofol is an intravenous anesthetic that has been widely used in clinics. Besides its anesthetic effects, propofol has also been reported to influence the regulation of the autonomic system. Controversies exist with regard to whether propofol exposure is safe for pregnant women and young children. In this work, human-induced pluripotent stem cell- (hiPSC-) derived neural progenitor cells (NPCs) were treated with propofol at 20, 50, 100, or 300 *μ*M for 6 h or 24 h, and acute and subacute cell injury, cell proliferation, and apoptosis were evaluated. Comparison of genome-wide gene expression profiles was performed for treated and control iPSC-NPCs. Propofol treatment for 6 h at the clinically relevant concentration (20 or 50 *μ*M) did not affect cell viability, apoptosis, or proliferation, while propofol at higher concentration (100 or 300 *μ*M) decreased NPC viability and induced apoptosis. In addition, 20 *μ*M propofol treatment for 6 h did not alter global gene expression. In summary, propofol treatment at commonly practiced clinical doses for 6 h did not have adverse effects on hiPSC-derived NPCs. In contrast, longer exposure and/or higher concentration could decrease NPC viability and induce apoptosis.

## 1. Introduction

Propofol (2,6-diisopropylphenol) is an anesthetic agent for induction and intravenous maintenance of anesthesia during surgery. It has also been used as a sedative agent in ICUs for diagnostic imaging tests such as MRIs and endoscopies. Besides its anesthetic effects, propofol has been reported to influence the regulation of the autonomic system [1–3]. Interestingly, although it has been widely used off-label for anesthesia maintenance in toddlers and pregnant women, propofol has not been approved by the FDA in either target population, probably partly due to safety concerns on the developing central nervous system [4, 5]. Some reports have linked general anesthetic use in rodent and nonhuman primate babies with induced widespread neuronal degeneration and/or apoptosis followed by long-term memory and learning deficiency in adults [6–14], while other reports have suggested that no such association exists [15]. These controversies could partly stem from the lack of recapitulative models that truly reflect the response of human brain cells to the treatment of general anesthetics agents including propofol.

Human neural progenitor cells (NPCs) have the potential to serve as an ideal in vitro system to evaluate the effect of propofol among other anesthetics agents [16–18]. However, human NPCs are usually derived from fetal brains, which are extremely difficult to obtain. In addition, human fetal brain-derived cells pose ethical concerns as well as exhibit interindividual variability due to the diverse genetic background of the sources and the age of the fetuses at the time of cell derivation. Human-induced pluripotent stem cells (hiPSCs) have recently emerged as a promising and convenient cell source for obtaining pure NPCs. hiPSCs are reprogrammed from somatic cells such as dermal fibroblasts with a cocktail of transcription factors, OCT4, SOX2, KLF4, and C-MYC [19, 20]. iPSCs can then be induced toward the neural lineage to give rise to NPCs and mature neural cells including neuron subtypes and glia. The use of NPCs derived from human iPSCs could theoretically provide a stable and inexhaustible cell source for in vitro testing of anesthetics. The NPCs have also become a platform for personalized medicine which could help determine the effects of certain anesthetics precisely for each and every individual patient tested.

In the current work, we attempted to examine whether propofol was toxic to hiPSC-derived NPCs. We found that propofol treatment at commonly practiced clinical doses for 6 h did not have adverse effects on hiPSC-derived NPCs. By a genome-wide gene expression analysis, we proposed several pathways that may be involved in the cytotoxicity of propofol at higher concentrations on multiple human NPC cell lines derived from iPSCs.

## 2. Materials and Methods

### 2.1. Cell Culture

Three hiPSC lines were used in this work. NESTIN-GFP knockin reporter (NES-GFP) and ND2-0 hiPSC were obtained from the NIH Center for Regenerative Medicine. hiPSC line USCK7 was generated in-house from human urine-derived cells by Cytotune (Life Technologies) reprogramming kit [21]. All hiPSCs were cultured on Matrigel-coated dishes in TeSR-E8 medium (Stemcell Technologies).

### 2.2. Generation of NPCs

NPCs were differentiated from hiPSCs following a modified dual SMAD inhibition method [22]. Briefly, hiPSCs were digested into small clumps using 0.5 mM EDTA, transferred to 10 cm Petri dishes and suspended in medium containing DMEM : F12, 20% knockout serum replacement, 1% nonessential amino acids (NEAA), 1% GlutaMAX, and 55 *μ*M 2-mercaptoethanol, supplemented with 10 *μ*M SB-431542 and 1 *μ*M dorsomorphin (Tocris). The medium was replaced on day 2 with neural induction (ND) medium containing DMEM : F12, 1% NEAA, 1% GlutaMAX, 1 mg/ml heparin, 1% N2, 1% B27, and 1% antibiotic-antimycotic solution, supplemented with 10 *μ*M SB-431542 and 1 *μ*M dorsomorphin. On day 6, the embryoid bodies were transferred to Matrigel-coated dishes and cultured in ND medium with 20 ng/ml basic fibroblast growth factor (bFGF). Within 3-4 days, typical neural rosettes were manually isolated under a dissection microscope and then treated with Accutase to form uniform NPCs. The NPCs were cultured and expanded in ND medium and passaged every 4-5 days with Accutase.

### 2.3. Propofol Treatment

Propofol (Sigma) was diluted with dimethyl sulfoxide (DMSO) to make a 500 mM stock solution. NPCs were treated at different concentrations (20, 50, 100, and 300 *μ*M) for 6 or 24 h.

### 2.4. MTT Assay

To detect early cell damage, MTT assay was performed according to manufacturer's instructions (Life Technologies). Briefly, NPCs were seeded on 96-well plates (3000 cells/well) and grown overnight. Cells were treated with propofol for 6 or 24 h, or 6 h followed by a 20 h washout period. MTT was added to the medium and the reaction was incubated at 37°C for 4 h. The MTT formazan product was then dissolved in DMSO and quantified spectrophotometrically at 540 nm.

### 2.5. Lactate Dehydrogenase (LDH) Assay

To detect late-stage cell damage, LDH assay was performed using LDH cytotoxicity assay kit (Thermo Scientific). Briefly, NPCs were seeded on 96-well plates and were treated with propofol (20, 50, 100, and 300 *μ*M) for 6 h. Fifty *μ*l of culture supernatant was harvested, mixed with equal volume of reaction mixture, and incubated at room temperature for 30 min. All samples were quantified spectrophotometrically at 490 nm and 680 nm.

### 2.6. Immunocytochemistry

Immunocytochemistry was performed as previously described [23]. Briefly, cells grown on glass coverslips were fixed with 4% paraformaldehyde and incubated in blocking buffer containing 5% goat serum, 1% bovine serum albumin, and 0.1% Triton X-100 for 30 min. Cells were incubated in primary antibodies at 4°C overnight. Appropriate Alexa Fluor secondary antibodies were used. Primary antibodies include Nestin (1 : 200, R&D systems), Sox1 (1 : 250, Millipore), and Ki-67 (1 : 500, Abcam). Nuclei were identified with DAPI (Sigma). Images were captured using a Zeiss AxioVision microscope with a z-stack split view function. For quantification of Ki-67+ cells, at least 1000 cells were counted for each staining.

### 2.7. Quantification of Apoptotic Cells

After propofol treatment, NPCs were labelled with the Annexin V-FITC apoptosis detection kit (BD) following manufacturer's instructions. Propofol-treated NPCs were suspended in Annexin V binding buffer at a concentration of 1 × 10^6^ cells/ml. Cell suspension (100 *μ*l) was mixed with 5 *μ*l of Annexin V-FITC and was incubated at room temperature for 15 min protected from light. Reaction was stopped with 400 *μ*l of Annexin V binding buffer. Dead cells were labelled with DAPI. The apoptosis ratio was measured by flow cytometry.

### 2.8. Microarray and DEG Analysis

Total RNA was isolated with RNAeasy mini kit (Qiagen) and run on HumanHT-12 v4 expression BeadChip kit (Illumina). BeadArray data was annotated with GenomeStudio. After variance-stabilizing transformation and normalization with the robust spline normalization method in the package lumi of *R*, differential analysis was performed using package limma for the following groups: (1) 20 *μ*M versus untreated control; (2) 300 *μ*M versus untreated. To screen for differentially expressed genes (DEGs), cut-off criteria were set as fold change (FC) > 2 (|Log FC| > 1) and *p* < 0.05. The expression value of DEGs from the 300 *μ*M treatment group and untreated was hierarchically clustered by package pheatmap of *R*. Raw and normalized data was submitted to Gene Expression Omnibus (GEO) under the accession number GSE101724.

### 2.9. Functional Enrichment Analysis

Functional enrichment analysis of DEGs was performed with DAVID (Database for Annotation, Visualization and Integrated Discovery) to identify gene ontology (GO) categories in biological processes and KEGG (Kyoto Encyclopedia of Genes and Genomes) signaling pathways. The false discovery rate (FDR) < 0.05 was set as the cut-off criterion.

### 2.10. PPI Network Construction

The protein-protein interaction (PPI) of the DEGs was obtained using STRING (search tool for the retrieval of interacting genes, https://string-db.org/) and visualized by the Cytoscape 3 software (http://www.cytoscape.org/). Combined score > 0.4 was set as the cut-off criterion for PPI relationship. The connectivity degree of each node of the network was calculated. Molecular COmplex DEtection (MCODE) was then used to find clusters based on topology to locate densely connected regions.

### 2.11. qRT-PCR

Total RNAs were extracted using Quick-RNA MiniPrep Kit (Zymo Research). RNA was converted to cDNA using the iScript cDNA synthesis kit (Bio-Rad). qRT-PCR was performed to determine mRNA levels using the iQ SYBR green supermix (Bio-Rad). GAPDH was used as an internal control. The relative fold change in gene expression was evaluated using the comparative threshold cycle ΔΔCt method. The qRT-PCR primers are listed in Supplementary Table S2 available online at https://doi.org/10.1155/2017/9182748.

### 2.12. Statistical Analysis

All data were represented as mean ± SD. All assays were repeated at least three times and each experiment was performed in triplicate. Data were analyzed using Student's *t*-test, where two independent groups were compared. *p* < 0.05 was considered to be significant.

## 3. Results

### 3.1. Generation of NPCs from hiPSCs

**Figure 1:**
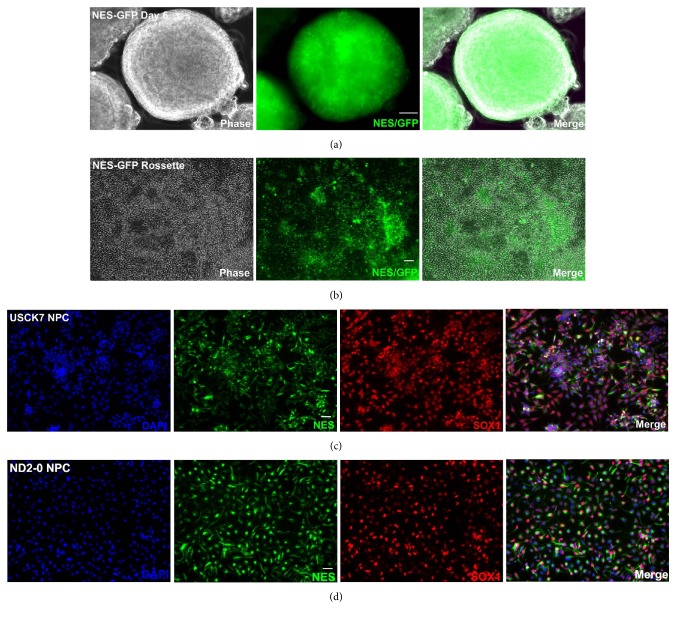
Generation of NPCs from hiPSCs. Representative images of neural tube structures generated from differentiating NES-GFP reporter hiPSC line via embryoid body formation method on day 6. GFP serves as a surrogate marker for NESTIN, a widely accepted NPC marker (a). The neural rosettes were attached to culture plates on day 10 as monolayer culture which continued to express GFP (NESTIN) (b). Similarly, NESTIN and another NPC marker SOX1 were both expressed robustly and uniformly in NPCs that were derived from two additional hiPSC lines, USCK7 (c) and ND2-0 (d), as revealed by immunocytochemistry staining of both NESTIN (green) and SOX1 (red). DAPI (blue) was used to reveal nuclei. Bar, 50 *μ*m.

The NPCs were generated with a modified dual SMAD inhibition method [22]. A good proportion of cells started to express Nestin as early as day 6 of differentiation, as indicated by GFP expression in the NES-GFP reporter (Figure 1(a)), and by NESTIN and SOX1 staining (Figures 1(c) and 1(d)). After manual isolation of neural rosettes (Figure 1(b)), pure NESTIN+/SOX1+ NPCs were obtained (Figures 1(c) and 1(d)).

### 3.2. Propofol Did Not Have Neurotoxicity to hiPSC-Derived NPCs at Clinically Relevant Concentrations

**Figure 2:**
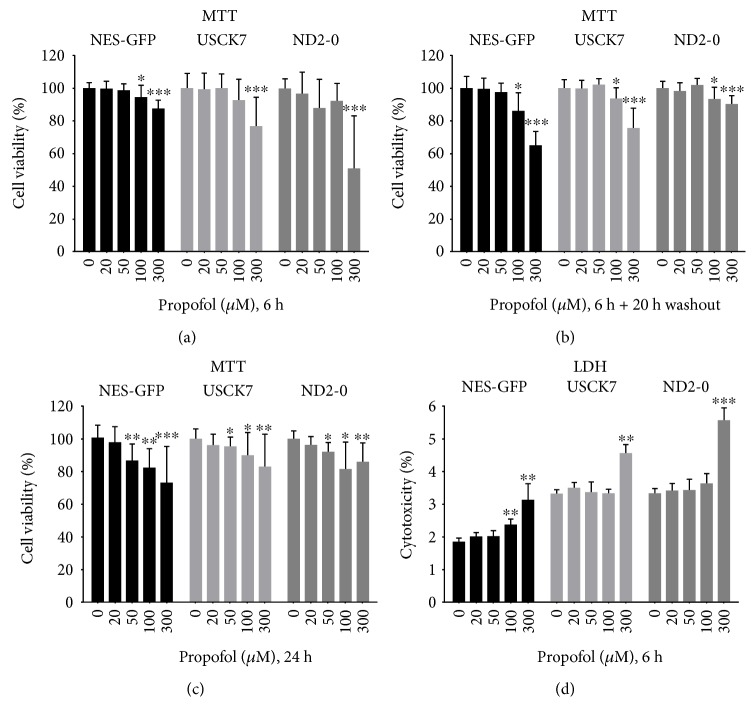
High-concentration propofol treatment exerts toxicity to NPCs. NPCs derived from three hiPSC lines were exposed to propofol at different concentrations (0, 20, 50, 100, and 300 *μ*M). MTT assays showed high-concentration propofol-reduced NPCs viability after the 6 h (a) or 24 h (c) treatment, or 6 h treatment followed by 20 h washout (b). Data from LDH assays further showed that propofol at a high concentration reduced the number of NPCs (d). Data at each concentration were analyzed by unpaired *t*-tests. For MTT assays, data were expressed as a percentage of viable cells of the treatment compared to the untreated (0 *μ*M propofol) group (mean ± SD). For LDH assays, data were expressed as a percentage of positive control (mean ± SD), compared to 0 *μ*M propofol exposure group. For both assays, 5 wells per treatment condition were examined. Each experiment was repeated for at least three times. ^∗^*p* < 0.05; ^∗∗^*p* < 0.01; ^∗∗∗^*p* < 0.001.

Previous reports indicated that the concentration of propofol in the brain during induction and maintenance of anesthesia is less than 10 *μ*g/ml (50 *μ*M) [24–26]. To determine the dosage of propofol treatment on NPCs, we chose 20, 50, 100, and 300 *μ*M. NPCs treated with propofol at 20 or 50 *μ*M for 6 h did not show any change in cell viability or late-stage cell injury as evaluated by MTT assay and LDH release (Figure 2), while NPCs treated with 300 *μ*M propofol for 6 h showed significantly decreased cell viability (*p* < 0.01) and induced cytotoxicity in all three cell lines, especially in NPCs derived from ND2-0 hiPSC line (Figure 2). Although propofol at 20 or 50 *μ*M did not show any toxicity at 6 h, after 24 h of treatment, the 50 *μ*M group showed a decrease in NPC viability in all cell lines (Figure 2), indicating that sustained exposure of propofol could result in negative effects on cell viability.

### 3.3. Propofol Did Not Induce Apoptosis in Human NPCs

**Figure 3:**
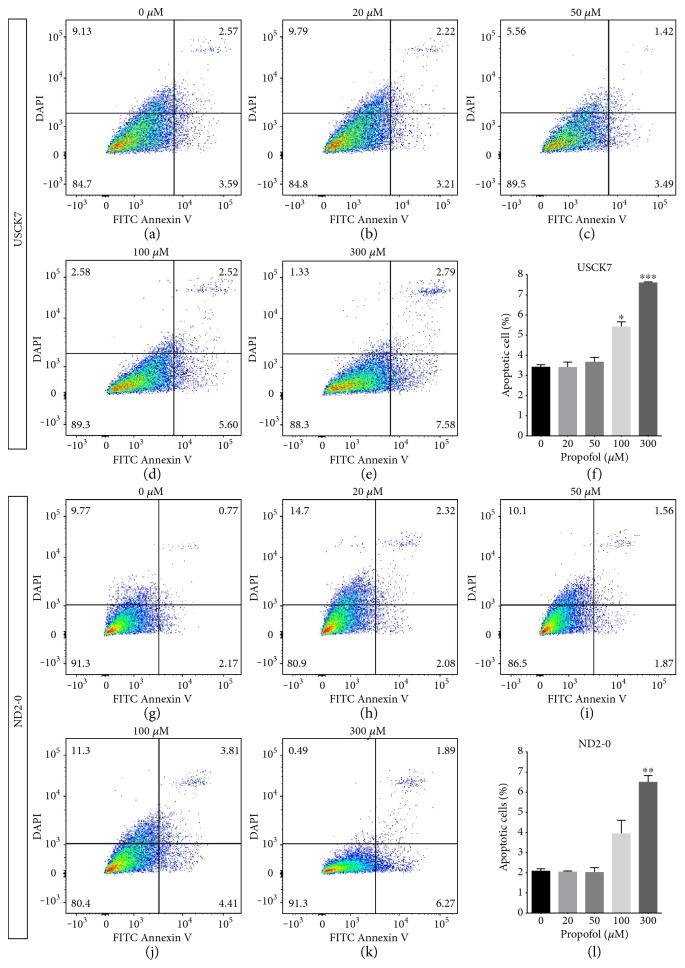
Propofol-induced apoptosis in NPCs. NPCs derived from USCK7 and ND2-0 were treated with propofol at 0, 20, 50, 100, and 300 *μ*M for 6 h and apoptotic cells quantified by flow cytometry of FITC-labelled Annexin V. The percentage of different cell populations is shown in each of the four quadrants of the representative flow cytometry charts, and the statistical analyses are summarized in (USCK7) (f) and (ND2-0) (i). The percentage of apoptotic cells is shown at the left lower quadrant of each chart. For USCK7-NPCs, the 100 and 300 *μ*M propofol treatment groups showed significantly higher percentage of apoptotic cells (a, b, c, d, e, f). For ND2-0 NPCs, only the 300 *μ*M treatment group showed a significantly higher percentage of apoptotic cells (g, h, i, j, k, l). Data were expressed as a percentage of FITC+/DAPI− cells (mean ± SD) for *n* = 3 flow cytometry experiments per treatment condition. ^∗^*p* < 0.05; ^∗∗^*p* < 0.01; ^∗∗∗^*p* < 0.001.

To investigate whether propofol could induce apoptosis in human NPCs, we treated NPCs with different concentrations of propofol for 6 h and quantified FITC-labeled Annexin V+ apoptotic cells by flow cytometry. Our results showed that exposure of NPCs to propofol at clinically relevant concentrations (20 or 50 *μ*M) for 6 h did not cause apoptosis in USCK7 or ND2-0 NPCs (Figures 3(a), 3(b), 3(c), 3(f), 3(g), 3(h), 3(i), and 3(l)). However, the percentage of apoptotic cells in both cell lines increased significantly after treatment with 300 *μ*M propofol (7.61 ± 0.03% versus 3.41 ± 0.1% in USCK7 and 6.5 ± 0.2% versus 2.1 ± 0.1% in ND2-0) (Figures 3(e), 3(f), 3(k), and 3(l)). The percentage of apoptotic cells also slightly increased in the 100 *μ*M propofol treatment group in both cell lines, although statistical significance could be found in ND2-0 NPCs only (Figures 3(d), 3(f), 3(j), and 3(l)).

### 3.4. Propofol Treatment Did Not Affect NPC Proliferation

**Figure 4:**
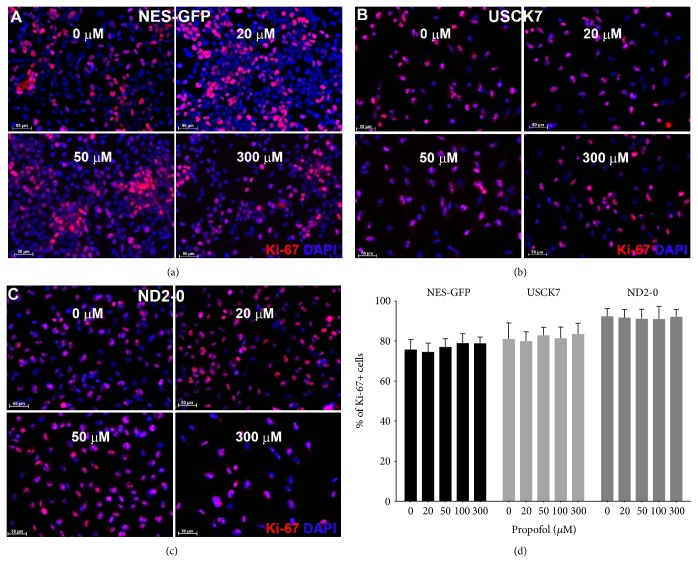
Propofol treatment for 6 h did not affect NPC proliferation. NPCs derived from three hiPSC lines were treated with propofol at different concentrations (0, 20, 50, 100, and 300 *μ*M). Cell proliferation was assessed by Ki-67 (red) immunocytochemistry staining. Nuclei were revealed by DAPI (blue) (a, b, c). At least 1000 cells were counted for each experiment. Data were expressed as percentage of Ki-67+ cells (mean ± SD). *n* = 3 Ki-67 staining per treatment condition. Bar, 50 *μ*m.

The percentage of Ki-67+ cells remained in the same range after treatment with different concentrations of propofol for 6 h in all three lines of NPCs (Figure 4).

### 3.5. Global Gene Expression Profiles of NPCs

**Figure 5:**
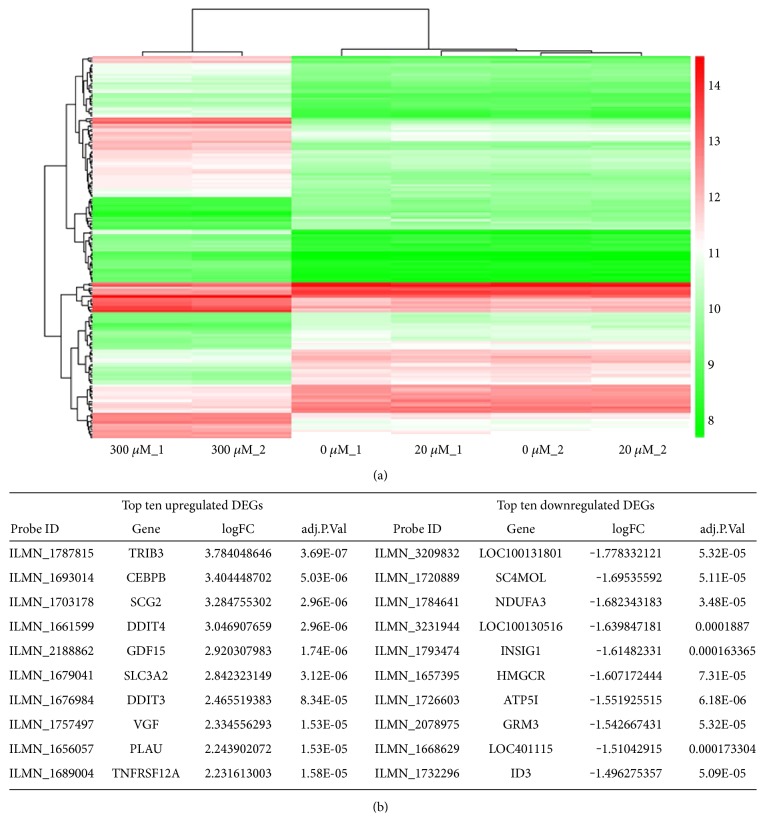
Gene expression profile of propofol-treated NPCs. NES-GFP iPSC-derived NPCs were treated with propofol (20 *μ*M or 300 *μ*M) for 6 h, and RNAs extracted immediately for Illumina BeadArray. Heatmap (a) of hierarchical clustering of differentially expressed genes (DEGs) shows that the 20 *μ*M propofol-treated group clustered together with the untreated group, while the 300 *μ*M propofol-treated group shows distinct gene expression profile. The top ten upregulated and downregulated DEGs are listed in (b).

Since propofol treatment at 300 *μ*M for 6 h significantly decreased cell viability and increased cytotoxicity and apoptosis in NPCs, we further examined global gene expression profiles and signaling pathways potentially involved in the effects of propofol on NPCs. Twenty *μ*M and 300 *μ*M were chosen for further comparison and analysis. No differentially expressed genes (DEGs) that satisfy our cut-off criteria (FC > 2, *p* < 0.05) were found between the 20 *μ*M and the untreated groups, indicating that propofol at 20 *μ*M did not alter gene expression of NPCs, which was further confirmed by the heatmap generated by hierarchical clustering analysis (Figure 5(a)), in which duplicates of untreated (0 *μ*M) and 20 *μ*M groups clustered together with completely indistinguishable gene expression patterns. On the other hand, the 300 *μ*M group showed a distinct gene expression pattern (Figure 5(a)) that clearly separated it from the untreated and the 20 *μ*M cluster. Further analysis revealed a total of 176 DEGs between the 300 *μ*M and the untreated control group, including 109 upregulated and 67 downregulated genes. The top 10 upregulated and downregulated DEGs are listed in Figure 5(b). Collectively, these analyses indicated that propofol at 20 *μ*M, 6 h, does not interfere with the gene expression of human NPCs.

### 3.6. Functional Enrichment Analysis

To further dissect the molecular changes in gene expression that might be caused by the treatment of a high-concentration propofol (300 *μ*M, 6 h), we performed functional annotation enrichment analysis of DEGs with bioinformatics tool DAVID (Database for Annotation, Visualization and Integrated Discovery, version 6.8, https://david.ncifcrf.gov/) to identify Gene Ontology (GO) categories in biological processes and KEGG signaling pathways. The false discovery rate (FDR) < 0.05 was set as the cut-off criterion. Fourteen biological process (BP) terms and 5 molecular function (MF) terms were obtained from the upregulated DEGs, and 7 cellular component (CC) terms were found from the downregulated DEGs. One KEGG pathway was identified from the upregulated DEGs and the 1 KEGG pathway was found from the downregulated DEGs (Supplementary Table S1). The upregulated DEGs were significantly related to protein translation and apoptosis regulation, while the downregulated DEGs were significantly related to mitochondrial function and oxidative phosphorylation.

### 3.7. PPI Network Construction Reveals Interaction of Critical Genes

**Figure 6:**
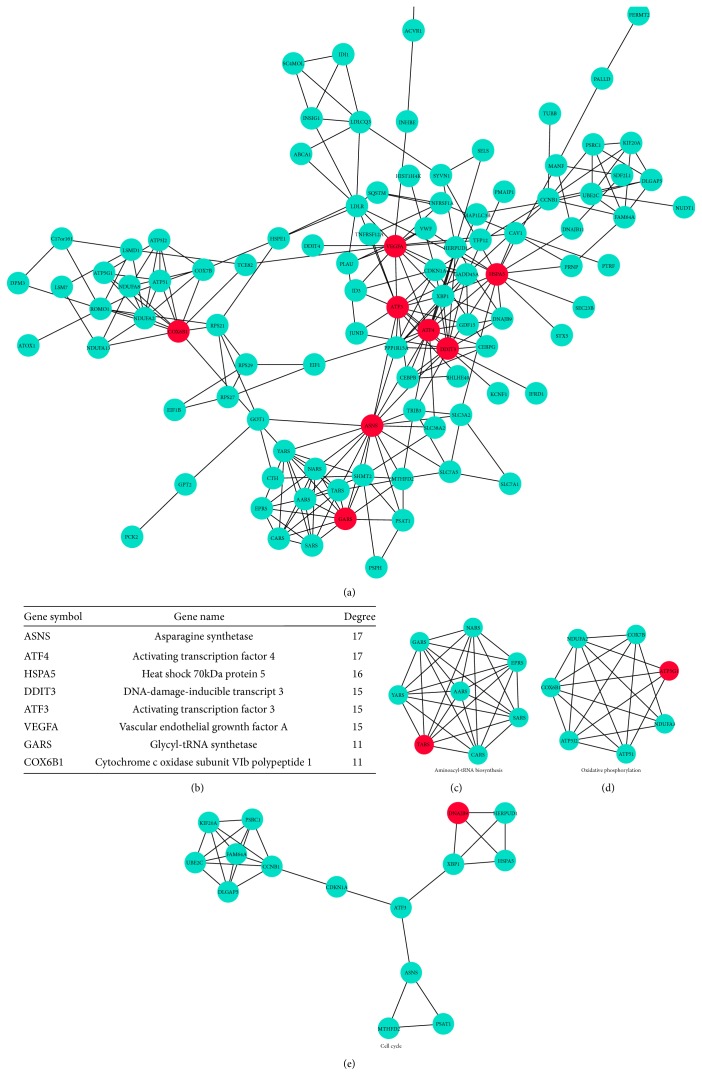
PPI network construction of DEGs extracted from comparison of treated (300 *μ*M) with untreated NPCs. The PPI relationships of the DEGs were obtained by using search tool for the retrieval of interacting genes (STRING) and visualized using the Cytoscape 3 software. After filtering out disconnected DEGs, a network with 101 nodes and 251 edges were obtained with the combined score > 0.4 (a). The connectivity degree of each node of the PPI network was calculated. Eight genes with connectivity degree > 10 were selected as hub genes (b). Clusters with densely connected regions based on topology were built with Molecular COmplex DEtection (MCODE). The top three subnetworks represent important cellular and molecular pathways including aminoacyl-tRNA biosynthesis (c), oxidative phosphorylation (d), and cell cycle (e).

**Figure 7:**
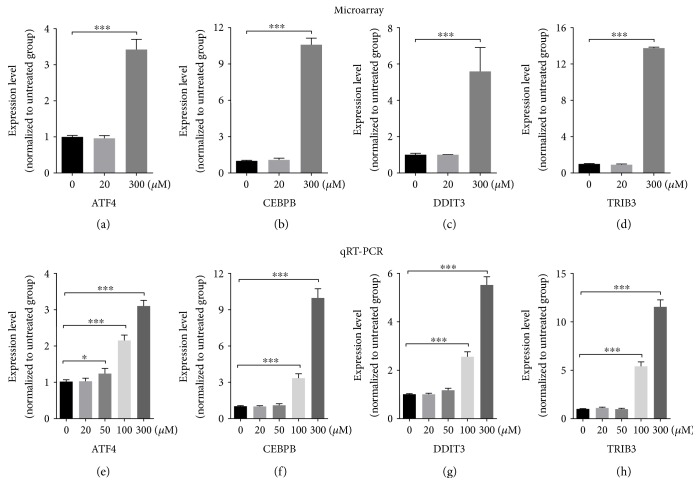
Verification of the expression level of genes related to UPR and ER press. NES-GFP iPSC-derived NPCs were treated with propofol (0, 20, 50, 100, or 300 *μ*M) for 6 h. The mRNA expression level of ATF4, CEBPB, DDIT3, and TRIB3 was evaluated by microarray (a, b, c, d) and qRT-PCR (e, f, g, h). The qRT-PCR results were normalized to the GAPDH mRNA level. All data are presented as mean ± SD (*n* = 3; ^∗^*p* < 0.05; ^∗∗∗^*p* < 0.001, Student's *t*-test).

After filtering out disconnected DEGs, a PPI network with 101 nodes (genes) and 251 edges (connections) was obtained with a combined score of >0.4 (Figure 6(a)). The connectivity degree of each node of the network was calculated. Eight genes with a connectivity degree > 10 were selected as hub genes (Figure 6(b)), which included critical molecules in ER stress-UPR signaling pathway such as ATF3, ATF4, DDIT3, and HSPA5. To better identify the hierarchy of critically involved genes and to determine densely connected regions, we performed an analysis with MCODE algorithm, aiming to find gene clusters based on topology. Seven subnetworks formed within the general DEG network, of which genes responsible for aminoacyl-tRNA biosynthesis, oxidative phosphorylation, and cell cycle were identified (Figures 6(c), 6(d), and 6(e)). We also verified the mRNA expression level of related genes by qRT-PCR. Expression of ATF4, CEBPB, DDIT3, and TRIB3 was significantly upregulated in the high-concentration propofol-treated group (300 *μ*M, 6 h), which is consistent with data extracted from microarray (Figure 7).

## 4. Discussion

In the current work, we assessed the effects of propofol at a clinically relevant and experimentally high dosage in hiPSC-derived NPCs for the first time. Our results showed that at clinical concentrations (20 and 50 *μ*M) and durations (6 h), propofol had no negative effects on human NPCs, while at higher concentrations (300 *μ*M) and durations (24 h), propofol induced apoptosis in NPCs. Our global gene expression analysis indicated that sustained endoplasmic reticulum (ER) stress and inhibition of mitochondrial oxidative phosphorylation are two major pathways that propofol might employ to execute its toxicity to hiPSC-derived NPCs. Aberration of both pathways would also lead to abnormal protein translation and energy metabolism in these cells.

Increasing concerns have recently arisen about the safe use of propofol in expecting mothers and young children, as the brain is thought to be vulnerable to anesthetics from the third trimester to the first 3 years of life [8, 11, 27, 28]. Animal models and cells derived from human fetal tissues have been used to mimic the developing brain. However, animal models do not always recapitulate human conditions especially in the case of CNS. Fetal tissues suffer from limited availability as well as interindividual genetic differences. These limitations have prompted us to search for alternative yet authentic human cell models, such as using the increasingly powerful hiPSCs and their various neural lineage derivatives.

Two types of human pluripotent stem cells, embryonic stem cells (hESCs) and induced pluripotent stem cells (hiPSCs), have been widely used. hESCs [29] are derived from the inner cell mass of blastocysts and theoretically have the potential to give rise to any lineage of the body. hiPSCs are reprogrammed from somatic cells and share a remarkable degree of similarity with hESCs on key cellular features, including genetic and epigenetic profiles, self-renewal capabilities, and differentiation potentials. Like hESCs, hiPSCs are able to generate cells of all three germ layers, including cells of the neuroectoderm. One of the most attractive advantages for hiPSCs over hESCs is that hiPSCs retain all genetic information of the individuals they are derived from and are therefore an autologous and personalized cell source. hiPSC-derived NPCs have the potential to faithfully represent the developing brain and could serve as an in vitro platform for drug screening and testing.

Here, we assessed the effects of propofol on multiple lines of hiPSC-NPCs. Our data are consistent with previous reports on propofol treatment on rat or human embryonic neural stem cells [9, 18, 30] and collectively suggested that short-term exposure (<6 h) of propofol within the commonly practiced clinical dose range is safe to human NPCs, while prolonged exposure could result in extensive toxic effects.

Multiple pathways have been reported to be involved in propofol neurotoxicity, including increasing calcium influx to trigger the caspase cascade in apoptosis and activating the GABAA receptor and the p75 neurotrophin receptor, leading to ATP depletion. Propofol could also downregulate miR-21, a microRNA proposed to be neuroprotective [30–33]. To comprehensively interrogate gene expression changes after propofol treatment, we performed microarray analysis, aiming to identify differentially regulated genes and protein-protein interactions (PPI) involved upon exposure to propofol. The upregulated genes indicated that propofol treatment could induce apoptosis and cell death in human NPCs through unfolded protein response- (UPR-) associated ER stress [34–36]. Previously identified UPR-related genes, such as DDIT3 (DNA damage-inducible transcript 3), TRB3 (tribbles-related protein 3), CEBP/*β*, GADD34, and ATF4, were all upregulated significantly in the 300 *μ*M group, indicating that the UPR pathway might participate in propofol-induced toxicity (Supplementary Table S1). As ER stress and the UPR pathway are highly likely involved in propofol toxicity, efforts on identifying potential therapeutic candidates that reverse such process could be helpful to maintain cellular homeostasis under high concentrations of propofol. For example, O-demethyldemethoxycurcumin, a curcumin analogue, has been shown to downregulate the expression of several ER stress signaling molecules, including PERK, IRE-1, and CHOP, and has neuroprotective effect against ER stress-induced cell death [37]. Among the downregulated genes, NDUFA3, NDUF2, ATP5I, ATP5J2, ATP5G1, and COX7B are related to mitochondrial oxidative phosphorylation. Molecules that boost mitochondrial oxidative phosphorylation could potentially alleviate propofol toxicity.

It is interesting to note that propofol treatment reduced cell viability in NPCs at high concentration (100 and 300 *μ*m) as shown by the MTT assays (Figure 2). However, the proportion of cell viability reduction in the MTT assays was much larger than the proportion of increased apoptosis as detected by Annexin V in flow cytometry (Figure 3). This discrepancy suggested that propofol exposure may cause cell cycle arrest followed by attenuated proliferation. We examined genes involved in the cell cycle progression of NPCs in response to propofol treatment. Our data showed that several cell cycle related genes, such as DDIT3, PPP1R15A, and GADD45A, were upregulated in the propofol-treated group, while other genes that are involved in cell cycle progression, including CCNA2, CDKN3, CDC2, and CDC25B, were downregulated. Additional experiments to elucidate the mechanism that cell cycle molecules are affected by propofol treatment are warranted.

There are some weaknesses in our study. Our work is an in vitro study. Although we identified candidate genes and potential mechanisms that could contribute to propofol toxicity, whether these candidates play a role in the clinics still needs to be rigorously tested in in vivo settings. These in vivo tests can be carried out using transgenic mice with perturbed genes identified from our hiPSC-NPC work presented here or treating mice with candidate molecules (e.g., the aforementioned O-demethyldemethoxycurcumin) that might lead to reduced propofol toxicity. In addition, While NPCs to a certain extent represent the fetal developing brain, tests with additional cell types including more differentiated neural cells and neuron subtypes would provide a more comprehensive picture on the impact of propofol. The recently emerged technique of generating cerebral organoids from hiPSCs [38, 39] will be able to provide multiple brain cell types in a 3D mini brain scenario; hence, testing the effects of propofol on the cerebral organoids will likely yield data of more translational relevance and mechanistic insights. In addition, hiPSCs can be differentiated into cells of the peripheral nervous system and cardiomyocytes, among other cell types. Our platform of using hiPSCs to examine the effects of propofol can be extended to the field of autonomic dysregulation. These experiments are ongoing.

In conclusion, our work showed that propofol treatment at clinical concentrations had no adverse effects on multiple hiPSC line-derived NPCs. At supraclinical dose, its toxicity is possibly exerted through the ER stress pathway and the disturbance of mitochondrial energy metabolism.

## Supplementary Material

Supplementary Table S1. Functional enrichment analysis for the up- and down-regulated DEGs. Supplementary Table S2. qPCR primers used in this work.
