# Supplementary material for: Effects of Propofol Treatment in Neural Progenitors Derived from Human-Induced Pluripotent Stem Cells
Source: Neural Plast. 2017 Oct 8;2017:9182748. doi: 10.1155/2017/9182748 (PMC5651106; doi:10.1155/2017/9182748)
Supplement: Supplementary file 1 — Supplementary Table S1. Functional enrichment analysis for the up- and down-regulated DEGs. Supplementary Table S2. qPCR primers used in this work. [file 9182748.f1.docx]

**Supplementary Table S1. Functional enrichment analysis for the up- and down-regulated DEGs**

| **Up-regulated DEGs** |  | **Term** | **Count** | **FDR** |
| --- | --- | --- | --- | --- |
|  | **GO TERM_BP_FAT** | GO:0034976~response to endoplasmic reticulum stress | 8 | 3.36E-06 |
|  |  | GO:0043038~amino acid activation | 8 | 3.13E-05 |
|  |  | GO:0043039~tRNA aminoacylation | 8 | 3.13E-05 |
|  |  | GO:0006418~tRNA aminoacylation for protein translation | 8 | 3.13E-05 |
|  |  | GO:0006984~ER-nuclear signaling pathway | 6 | 0.005 |
|  |  | GO:0043066~negative regulation of apoptosis | 13 | 0.006 |
|  |  | GO:0043069~negative regulation of programmed cell death | 13 | 0.007 |
|  |  | GO:0060548~negative regulation of cell death | 13 | 0.007 |
|  |  | GO:0042981~regulation of apoptosis | 19 | 0.007 |
|  |  | GO:0043067~regulation of programmed cell death | 19 | 0.008 |
|  |  | GO:0010941~regulation of cell death | 19 | 0.009 |
|  |  | GO:0034620~cellular response to unfolded protein | 5 | 0.016 |
|  |  | GO:0030968~endoplasmic reticulum unfolded protein response | 5 | 0.016 |
|  |  | GO:0006399~tRNA metabolic process | 8 | 0.021 |
|  | **GO TERM_MF_FAT** | GO:0004812~aminoacyl-tRNA ligase activity | 8 | 1.62E-05 |
|  |  | GO:0016875~ligase activity, forming carbon-oxygen bonds | 8 | 1.62E-05 |
|  |  | GO:0016876~ligase activity, forming aminoacyl-tRNA and related compounds | 8 | 1.62E-05 |
|  |  | GO:0046983~protein dimerization activity | 15 | 0.006 |
|  |  | GO:0015171~amino acid transmembrane transporter activity | 6 | 0.038 |
|  | **KEGG** | hsa00970:Aminoacyl-tRNA biosynthesis | 8 | 1.97E-05 |
| **Down-regulated DEGs** | **GO TERM_CC_FAT** | GO:0044455~mitochondrial membrane part | 7 | 0.001640054 |
|  |  | GO:0005743~mitochondrial inner membrane | 9 | 0.002421772 |
|  |  | GO:0019866~organelle inner membrane | 9 | 0.004152384 |
|  |  | GO:0031090~organelle membrane | 14 | 0.007429436 |
|  |  | GO:0031966~mitochondrial membrane | 9 | 0.015594093 |
|  |  | GO:0005740~mitochondrial envelope | 9 | 0.024335427 |
|  |  | GO:0044429~mitochondrial part | 10 | 0.043409398 |
|  | **KEGG** | hsa00190:Oxidative phosphorylation | 7 | 0.025 |

**Supplementary Table S2.** qPCR primers used in this work.

| **Gene symbol** | **Forward primer** | **Reverse primer** |
| --- | --- | --- |
| ATF4 | AGTGCATCTGTATGAGCCCA | GCTCCTATTTGGAGAGCCCCT |
| CEBPB | CTTCAGCCCGTACCTGGGAC | GGAGAGGAAGTCGTGGTGC |
| DDIT3 | GGAAACAGAGTGGTCATTCCC | CTGCTTGAGCCGTTCATTCTC |
| TRIB3 | GCCCTGCACTGCCCTACAC | GGTACCAGCCAGGACCTCAGT |
